# Can Beta-2-Adrenergic Pathway Be a New Target to Combat SARS-CoV-2 Hyperinflammatory Syndrome?—Lessons Learned From Cancer

**DOI:** 10.3389/fimmu.2020.588724

**Published:** 2020-09-30

**Authors:** Antonio Barbieri, Nirmal Robinson, Giuseppe Palma, Nicola Maurea, Vincenzo Desiderio, Gerardo Botti

**Affiliations:** ^1^Animal Facility, Istituto Nazionale Tumori, Istituto Di Ricovero e Cura a Carattere Scientifico “Fondazione G. Pascale”, Naples, Italy; ^2^Centre for Cancer Biology, University of South Australia and SA Pathology, Adelaide, SA, Australia; ^3^Division of Cardiology, Istituto Nazionale Tumori, Istituto Di Ricovero e Cura a Carattere Scientifico (IRCCS) “Fondazione G. Pascale”, Naples, Italy; ^4^Section of Histology, Department of Experimental Medicine, University of Campania “Luigi Vanvitelli”, Naples, Italy; ^5^Scientific Directorate, Istituto Nazionale Tumori, IRCCS “Fondazione G. Pascale”, Naples, Italy

**Keywords:** COVID-19, SARS-CoV2, Beta adrenergic receptors, beta-blockers, Cytokine storm, immune response, hyperinflammation

## Abstract

SARS-CoV-2 infection is a new threat to global public health in the 21^st^ century (2020), which has now rapidly spread around the globe causing severe pneumonia often linked to Acute Respiratory Distress Syndrome (ARDS) and hyperinflammatory syndrome. SARS-CoV-2 is highly contagious through saliva droplets. The structural analysis suggests that the virus enters human cells through the ligation of the spike protein to angiotensin-converting enzyme 2 (ACE_2_). The progression of Covid-19 has been divided into three main stages: stage I—viral response, stage II—pulmonary phase, and stage III—hyperinflammation phase. Once the patients enter stage III, it will likely need ventilation and it becomes difficult to manage. Thus, it will be of paramount importance to find therapies to prevent or slow down the progression of the disease toward stage III. The key event leading to hyperinflammation seems to be the activation of Th-17 immunity response and Cytokine storm. B_2_-adrenergic receptors (B_2_ARs) are expressed on airways and on all the immune cells such as macrophages, dendritic cells, B and T lymphocytes. Blocking (B_2_AR) has been proven, also in clinical settings, to reduce Th-17 response and negatively modulate inflammatory cytokines including IL-6 while increasing IFN*γ*. Non-selective beta-blockers are currently used to treat several diseases and have been proven to reduce stress-induced inflammation and reduce anxiety. For these reasons, we speculate that targeting B_2_AR in the early phase of Covid-19 might be beneficial to prevent hyperinflammation.

## Background

Coronaviruses are a variable group of enveloped, positive-sense, single-stranded RNA viruses (1). They cause several diseases involving respiratory, enteric, hepatic, and neurological systems with high severity among humans and animals (1, 2). In the last two decades, the entire world witnessed two novel types of coronavirus, severe acute respiratory syndrome CoV (SARS-CoV) and Middle East respiratory syndrome CoV (MERS-CoV), causing severe human diseases (3, 4). In December 2019 an outbreak of pneumonia of unknown cause occurred in Wuhan, Hubei Province, China that rapidly spread around the globe, reaching to pandemic dimensions, and it was later named as SARS-CoV-2 or COVID-19 (5, 6). The main symptoms of COVID-19 include fever, fatigue, and cough, which are similar to that of SARS-CoV and MERS-CoV infected cases. SARS-CoV-2 is closely related to other coronaviruses (88% identity) which likely originated from bats. SARS-CoV-2 enters the human cells through ACE_2_ receptors after ligation of spike protein. These receptors are highly expressed in the lung but are also found in other organs such as the heart, kidney, endothelium, and intestine (7–9). Notably, ACE_2_ is highly expressed on the luminal surface of intestinal epithelial cells, functioning as a co-receptor for nutrient uptake, in particular for amino acid resorption from food (10). For this reason, the intestine might also be a putative entry site for SARS-CoV-2 and that the infection might have been initiated by eating food from wild animals. To date the virus has infected hundreds of thousands of people, and about half of the hospitalized patients show clinical signs of comorbidities such as hypertension (23.7–30%), diabetes mellitus (16.2%), coronary heart diseases (5.8%), and cerebrovascular disease (2.3%). Also, a high rate of mortality has been reached when one or more comorbidities coexist. Covid-19 infection associated symptoms, including acute respiratory distress syndrome (ARDS) and septic shock seem to be associated with hyperinflammation and cytokine storm as in patients’ serum the levels of several cytokines are elevated (11). In particular IL-6, IL-1*β*, IL-10, TNF, GM-CSF, IP-10 (IFN-induced protein 10), IL-17,MCP-3, and IL-1ra are mainly involved both in mild and severe forms of disease and altered circulating leukocyte subset and cytokine secretion (12). As many of these cytokines are involved in the Th17 type response, Wu and Yang suggested that targeting the Th17 pathway may counteract the Covid-19 symptoms (13). This hypothesis relies on different pieces of evidence: IL-6, TNF*α*, and IL-1*β* promote Th17 response and are associated with inflammatory symptoms including fever, and the two latter are also associated with vascular permeability and leakage; IL-17 has a broad inflammatory effect and together with GM-CSF is involved in inflammatory and autoimmune disease; Covid-19 patients have a significantly increased number of CCR6+ Th17 cells (4) ; elevated TH17 and IL-17 related pathways are increased in SARS-CoV, MERS-CoV, and H1N1 influenza virus patients (14–16); In MERS-CoV patients, IL-17 and low IFN*γ* are associated with worse prognosis (14). Targeting beta-2-adrenergic pathway was shown to reduce inflammatory cytokine and Th17 response in different settings such as cancer and autoimmune diseases. For this reason we believe that beta-2-adrenergic pathway should be more deeply investigated as a possible target to reduce inflammation-related symptoms of SARS-CoV2.

## Beta-Adrenergic Receptors and Immune System

*β*-Adrenergic receptors are G-protein coupled transmembrane proteins. There are three subtypes of *β*-adrenergic receptors (*β*1-ARs, *β*2-ARs, and *β*3-ARs) that mediate a wide range of physiological responses to catecholamines epinephrine and norepinephrine, and thus play an important role in regulating cardiovascular responses in health and disease. *β*-Adrenoceptors regulate many aspects of airway function, including airway smooth muscle tone, mast cell mediator release, and plasma exudation. *β*1-adrenergic receptors are located mainly in the heart and in the kidneys (17, 18). *β*2-adrenergic receptors are located in the lungs, gastrointestinal tract, liver, uterus, vascular smooth muscle, and skeletal muscle (17, 18). *β*3-adrenergic receptors are located in fat cells. More than 90% of all *β*-receptors in the human lung are located in the alveoli where the *β*2-subtype predominates (70%) (19). However, *β*1- and *β*2-subtypes also coexist and are distributed uniformly in the alveolar walls. *β*2-adrenergic receptors are expressed by all the cells of the immune system, including T and B lymphocytes, dendritic cells (DCs) and macrophages (20). The specific role of adrenergic signaling in regulating immune responses and inflammation is still under debate. However, there are many pieces of evidence supporting a proinflammatory action and promotion of TH17 response. Manni et al. showed that triggering *β*2-adrenergic signal stimulates murine DC to secrete IL-6 and promote a Th17 response (21). In DC, the *β*2-adrenergic signal also reduces IL-12 and IFN*γ* production but promotes IL-17. *β*2-AR signaling plays a pivotal role in macrophage activation and proinflammatory cytokine production. Similarly, in other immune cells, the overall effect of *β*2-adrenergic stimulation is an exacerbation of inflammation, promotion of B cell antibody production, and stimulation of DC and macrophages to secrete proinflammatory cytokines. Very interestingly, Chiarella et al. showed that *β*2-AR on alveolar macrophages is responsible for IL-6 secretion and promotion of inflammation and prothrombotic state in a murine model of particulate-matter-induced thrombosis. Administration of *β*2-AR agonist induces mitochondria-dependent reactive oxygen species (ROS) generation which results in cAMP response element-binding protein (CREB) that results in IL-6 activation. Huang et al. investigated the effect of lymphocyte-derived catecholamines on the differentiation and function of T helper (Th) cells, suggesting that this shifted the Th1/Th2 balance in the direction of greater Th2 polarization (22, 23). Panina-Bordignon et al. also showed that beta2-adrenergic signaling inhibits the production of IL-12, thereby promoting Th2 differentiation and inhibiting Th1 development associated with antitumor immunity. Catecholamines are also known to impact the immune response *via* down-regulation of IFN-*γ* production (24). Functionally, IFN-*γ* can exert direct antiviral effects on infected cells as well as neighboring cells (25, 26). It can also activate local immune cells, like tissue-resident dendritic cells, macrophages, and NK cells, to augment antiviral functions (24, 27–29). Furthermore, IFN-*γ* can also control the antiviral state by modulating the differentiation and maturation of T cells and B cells (30, 31). Khalili et al. showed that propranolol, a non-selective beta-blocker, synergized with an HSP-70-rich tumor lysate vaccine to increase IFN-*γ* production in a murine model of fibrosarcoma (32). Treated animals showed lower rates of tumor growth (P < 0.01) and increased levels of CTL activity (P < 0.05).

## Beta-Blockers

Beta-blockers are a class of medications that are prevalently used to manage abnormal heart rhythms, hypertension and to protect the heart from recurring myocardial infarction. There are two main categories of beta-blockers: non-selective and selective. The first group includes older molecules such as propranolol, nandol, timol, *etc*., which have different degrees of affinity for *β*2-AR and *β*1-AR; second-generation drugs that are more selective such as metoprolol and acebutolol, *etc*. are specific for *β*1-AR. After the introduction of cardioselective beta-blockers, the non-selective are not used frequently to treat heart conditions as the selective ones have fewer side effects. However, they are still in use to treat several other conditions such as long QT syndrome, aortic dilation in Marfan syndrome, liver cirrhosis to reduce portal hypertension and bleeding esophageal varices.

Propranolol, a non-selective beta-adrenergic blocker, has been extensively used for over 50 years in the treatment of many cardiovascular problems such as ischemic heart diseases, arrhythmias, and heart malfunction. Recently, several clinical trials are supporting its benefit in a number of conditions, including cancer, hemorrhage, sepsis, and hypermetabolic syndrome associated with severe burns, akathisia associated with Alzheimer’s disease or psychosis, aggression associated with brain injury or disease, and anxiety (33–35). Two different reports on cancer patients show that propranolol treatment reduces inflammatory cytokines including IL-6 and TNF*α*, inflammation-related transcription factors such as NFκB and STAT3 and reduces the activation of Treg lymphocytes (36, 37). They also showed the potential benefit of propranolol on cancer recurrence (CR) and overall survival (OS) (36, 37). In these trials on breast cancer patients, propranolol treatment well-preserved the anticancer immunological profiles of peripheral blood mononuclear cells, reduced EMT and prometastatic and proinflammatory transcription factors in tumor samples. In addition, Shaashua and colleagues (38) have suggested a positive synergistic effect of anti-inflammatory and beta-blockers, when these drugs are co-administered.

Beta-adrenergic receptor antagonists are also known to have effects on platelet aggregation, and a meta-analysis published in 2014 showed that they decreased platelet aggregation by 13% (95% CI = 8–17%, standardized mean difference = −0.54, 95% CI = −0.85 to −0.24, P < 0.0001) (39). In particular non-selective lipophilic beta-blockers (including propranolol) decreased platelet aggregation more than that of the selective non-lipophilic beta-blockers. In addition, nonselective beta-blockers have been proved effective on the acute prothrombotic response to psychosocial stress and elevated plasma levels of factor VIII:C in patients with deep vein thrombosis (40, 41).

## Our Experience in Cancer

**Figure 1:**
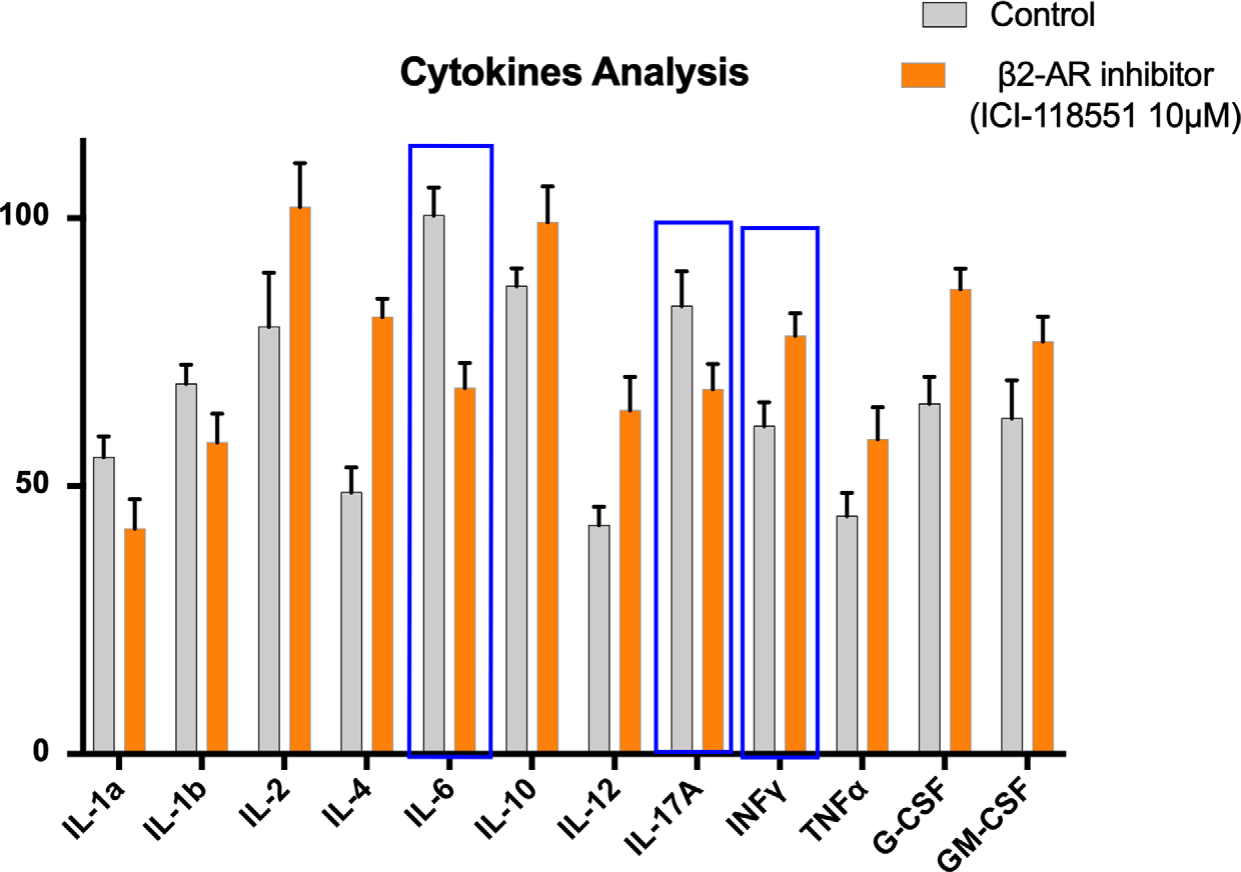
Proinflammatory cytokines in mice untreated (Control) or treated with beta-2-inhibitor (ICI115,881). 12 Cytokine Multiplex assay was performed through the quantification in serum of IL-1*α*, IL-1*β*, IL-2, IL-4, IL-6, IL-10, IL-12, IL17-*α*, IFN-*γ*, TNF-*α*, G-CSF, GM-CSF, in a lung colonization model of mice bearing murine melanoma cell line B16F10. Error bars depict means ± SD. One-way ANOVA and Bonferroni *post-hoc* analysis were used to examine the significance of differences among groups (Graph pad Prism 8.0). A probability value with P < 0.05 was considered to be statistically significant.

We had previously reported that propranolol reduces the effects of hypothalamic–pituitary–adrenal (HPA) axis stimulation induced by chronic stress. In particular, in a melanoma mouse model, propranolol treatment a week prior to the induction of stress delayed tumor growth. Furthermore, propranolol-treated mice showed lower levels of VEGF and eNOS with respect to untreated mice proving that it reduces the production of proangiogenic factors involved in tumor growth (42). Our reported findings also show that propranolol exerts antimetastatic effects on DU145 prostate cell lines xenograft in mice. It decreased metastatic foci in the inguinal lymph nodes, significantly reducing MMP2 and MMP9 in tumor samples. Moreover, a norepinephrine-dependent increase in epithelial to mesenchymal transition (EMT) can be rescued by propranolol treatment (43). Our preliminary data (**Figure 1**) in mouse lung colonization model of melanoma show a significative reduction of IL-6 and IL-17A levels and an increase of IFN-*γ* in mice treated with selective *β*2-AR inhibitor ICI 118,551 (ICI). Moreover, our data show an increase of IL-12 as shown in ICI-treated mice with respect to the controls.

## The Possible Benefit of *β*2-AR Targeting in Patients With COVID-19

The patients with SARS-CoV-2 at the late stages of the disease suffer from many abnormalities, which are the result of immune system imbalance and malfunction which can lead to proinflammatory reactions and immunopathological conditions, presented by lethal inflammation in the lungs and vascular leakage (44, 45). Several pieces of evidence support the pivotal role of IL-6 in driving these phenomena. Indeed, clinical evidence from China and Italy in different hospitals (46) showed that the IL-R6 blocker Tocilizumab, normally used for the treatment of rheumatoid arthritis can effectively improve clinical conditions of patients. The *β*2-AR blockade can reduce IL-6 and other inflammatory cytokines in the patient’s serum contributing to rebalancing the immune system. In 1980, Kaplan et al. suggested a possible role of propranolol in the treatment of rheumatoid arthritis (47). Moreover, the stress-induced inflammation, which likely occurs in patients diagnosed with Covid-19, could worsen the clinical symptoms; non-selective beta-blockers could prevent this phenomenon and at the same time reduce anxiety in those patients. Furthermore, the pericyte injury due to virus infection may result in capillary endothelial cell dysfunction, inducing microvascular dysfunction (48). Indeed, Tang et al. proved that anticoagulant treatment is associated with decreased mortality in severe Covid-19 patients with coagulopathy (49). Beta-blocker might counteract this by reducing prothrombotic response, vascular tone, and VEGF secretion (50–52). Notably, some authors demonstrated that propranolol inhibits choroidal neovascularization (CNV) *in vivo* and *β*2-AR blockade reduces vascular endothelial growth factor (VEGF) expression in mouse retinal pigment epithelium and choroidal endothelial cells in culture (53).

## Beta-Blockers and Respiratory Diseases

Since beta-blockers may cause bronchospasm, in some cases, their use is not recommended in Asthma or Chronic Obstructive Pulmonary Disease (COPD). However, a recent study on a large number of patients in Denmark showed that in patients taking beta-blockers (including non-selective) the risk of hospitalization for COPD is reduced (54). Similarly, in asthma, a recent systematic revision of the literature suggests that the escalating dose of beta-blockers is well tolerated and could be beneficial for airway inflammation and hyperresponsiveness (55). The patients positive for the SARS-CoV-2 did not describe any worsening of the symptoms. However, more accurate data are necessary to establish the safety of adrenergic targeting in these patients.

## Conclusions

**Figure 2:**
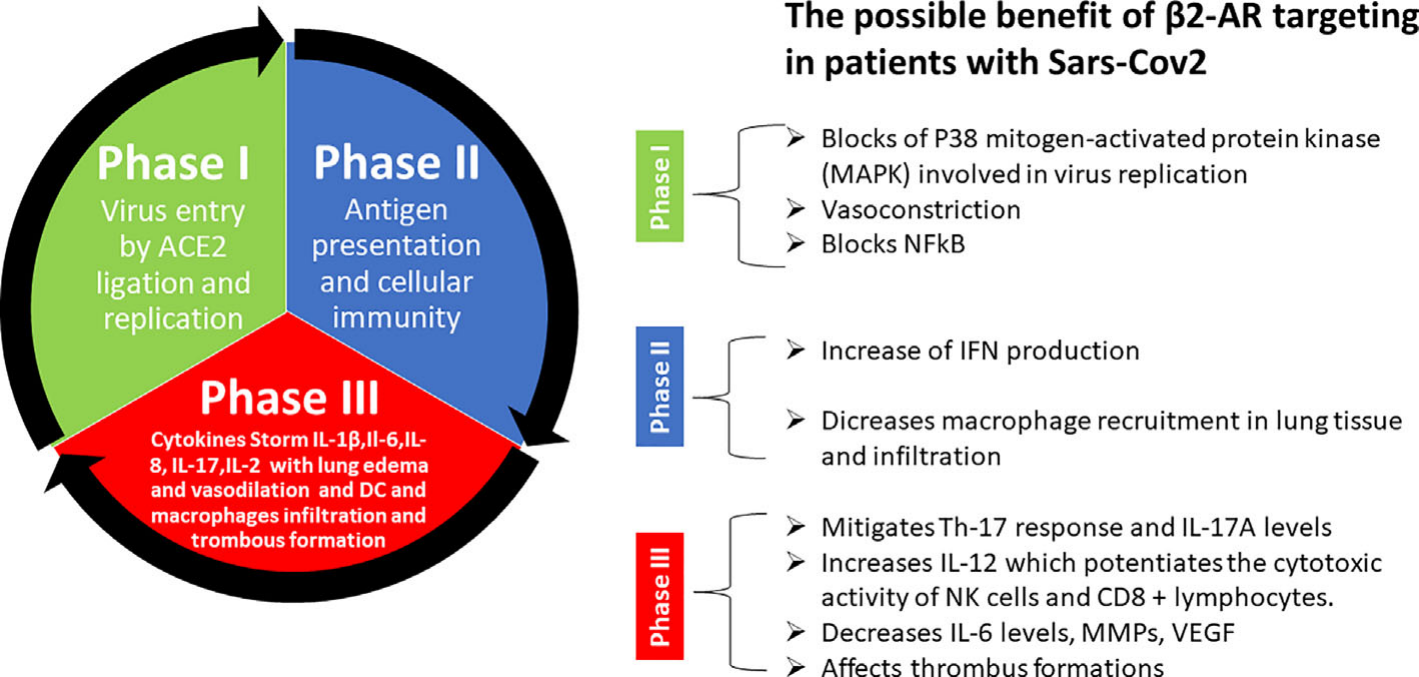
Summary of possible benefits of *β*2-AR targeting in patients with Sars-Cov-2. Explanation step by step of different phases of Sars-Cov-2 pathogenesis and possible tool offered by beta-blockers to prevent and counteract uncontrolled cytokine storm and thrombus formation in late phase III that lead to the death of patient.

Many supporting pieces of evidence show that the major symptoms of Covid-19 infection are associated with hyperinflammation over-activation of Th17 response. Indeed, treatments known to reduce Th17 response such as Tocilizumab and Ruxolitinib have been already used to treat Covid-19 patients in phases II and III. *β*2-AR signals have been described to have a central role in rheumatoid arthritis in promoting inflammation and Th17 response. Non-selective beta-blockers have been used in clinical settings to reduce inflammation and Th17 response. In addition, the non-selective beta-blocker propranolol blocks AT1 induced expression of IL-6, NFkB, TNF, IFN-*γ*, VEGF, and Metalloprotease Engagement. We believe that *β*2-AR signals might be a valuable target for new strategies aiming to block or slow down the transition from the early phases (I and IIa) of Covid-19 to phase III by reducing the activation of Th17 response and inflammatory cytokine release and prevent venous thromboembolism (**Figure 2**). For these reasons we would like to point out the importance to perform further preclinical and clinical studies to explore this opportunity.

## Data Availability Statement

The authors acknowledge that the data presented in this study must be deposited and made publicly available in an acceptable repository prior to publication. Frontiers cannot accept a manuscript that does not adhere to our open data policies.

## Author Contributions

AB and VD have thought and wrote the manuscript. NR critically revised the manuscript. GP revised immunological aspects and made corrections. NM made important contribution on beta-blockers. GB critically revised the manuscript and supervised the study. All authors contributed to the article and approved the submitted version.

## Funding

This work was funded by an “Ricerca Corrente” grant from the Italian Ministry of Health.

## Conflict of Interest

The authors declare that the research was conducted in the absence of any commercial or financial relationships that could be construed as a potential conflict of interest.
